# Social Deficits and Cerebellar Degeneration in Purkinje Cell *Scn8a* Knockout Mice

**DOI:** 10.3389/fnmol.2022.822129

**Published:** 2022-04-26

**Authors:** Xiaofan Yang, Hongqiang Yin, Xiaojing Wang, Yueqing Sun, Xianli Bian, Gaorui Zhang, Anning Li, Aihua Cao, Baomin Li, Darius Ebrahimi-Fakhari, Zhuo Yang, Miriam H. Meisler, Qiji Liu

**Affiliations:** ^1^Department of Pediatrics, Qilu Hospital of Shandong University, Jinan, China; ^2^Key Laboratory of Experimental Teratology, Ministry of Education, Department of Genetics, School of Basic Medical Sciences, Shandong University, Jinan, China; ^3^Medical School, State Key Laboratory of Medicinal Chemical Biology, Key Laboratory of Bioactive Materials for Ministry of Education, Nankai University, Tianjin, China; ^4^Department of Operational Medicine, Tianjin Institute of Environmental & Operational Medicine, Tianjin, China; ^5^Department of Neurology, Second Hospital of Shandong University, Jinan, China; ^6^Department of Radiology, Qilu Hospital of Shandong University, Jinan, China; ^7^Department of Neurology, The F.M. Kirby Neurobiology Center, Boston Children's Hospital, Harvard Medical School, Boston, MA, United States; ^8^Department of Human Genetics, University of Michigan, Ann Arbor, MI, United States; ^9^Department of Neurology, University of Michigan, Ann Arbor, MI, United States; ^10^Key Laboratory of Birth Regulation and Control Technology of National Health Commission of China, Maternal and Child Health Care Hospital of Shandong Province, Jinan, China

**Keywords:** Purkinje cell, *SCN8A*, mouse, autism, cerebellum, anxiety

## Abstract

Mutations in the *SCN8A* gene encoding the voltage-gated sodium channel α-subunit Nav1. 6 have been reported in individuals with epilepsy, intellectual disability and features of autism spectrum disorder. *SCN8A* is widely expressed in the central nervous system, including the cerebellum. Cerebellar dysfunction has been implicated in autism spectrum disorder. We investigated conditional *Scn8a* knockout mice under C57BL/6J strain background that specifically lack *Scn8a* expression in cerebellar Purkinje cells (*Scn8a*^*flox*/*flox*^, *L7Cre*^+^ mice). Cerebellar morphology was analyzed by immunohistochemistry and MR imaging. Mice were subjected to a battery of behavioral tests including the accelerating rotarod, open field, elevated plus maze, light-dark transition box, three chambers, male-female interaction, social olfaction, and water T-maze tests. Patch clamp recordings were used to evaluate evoked action potentials in Purkinje cells. Behavioral phenotyping demonstrated that *Scn8a*^*flox*/*flox*^, *L7Cre*^+^ mice have impaired social interaction, motor learning and reversal learning as well as increased repetitive behavior and anxiety-like behaviors. By 5 months of age, *Scn8a*^*flox*/*flox*^, *L7Cre*^+^ mice began to exhibit cerebellar Purkinje cell loss and reduced molecular thickness. At 9 months of age, *Scn8a*^*flox*/*flox*^, *L7Cre*^+^ mice exhibited decreased cerebellar size and a reduced number of cerebellar Purkinje cells more profoundly, with evidence of additional neurodegeneration in the molecular layer and deep cerebellar nuclei. Purkinje cells in *Scn8a*^*flox*/*flox*^, *L7Cre*^+^ mice exhibited reduced repetitive firing. Taken together, our experiments indicated that loss of *Scn8a* expression in cerebellar Purkinje cells leads to cerebellar degeneration and several ASD-related behaviors. Our study demonstrated the specific contribution of loss of Scn8a in cerebellar Purkinje cells to behavioral deficits characteristic of ASD. However, it should be noted that our observed effects reported here are specific to the C57BL/6 genome type.

## Introduction

Autism spectrum disorder (ASD) is a behaviorally defined pervasive neurodevelopmental disorder, characterized by persistent impairment of social communication, restricted interests and repetitive behaviors (Varghese et al., 2017). In addition to these core symptoms, there may be psychiatric or neurological comorbidities, of which attention-deficit/hyperactivity disorder (ADHD), anxiety, depression and epilepsy are most common (Lord et al., 2020). It is estimated that the prevalence of ASD is 1–2% in the general population (Wisniowiecka-Kowalnik and Nowakowska, 2019). Over the past decade, genomic technologies have enabled rapid progress in the identification of genes linked to ASD (Abrahams and Geschwind, 2008; Arnett et al., 2019). Although much effort has centered on the genetic delineation of syndromic forms of ASD, the underlying molecular mechanisms remain incompletely understood (Hampson and Blatt, 2015).

Several lines of evidence implicate cerebellar dysfunction in the development of ASD. Post-mortem studies have demonstrated a reduced number and density of cerebellar Purkinje cells (PC) in patients with ASD, and isolated cerebellar injury has been associated with a higher incidence of ASD (Wang et al., 2014; Hampson and Blatt, 2015). The cerebellum has been consistently implicated in several monogenetic syndromes associated with ASD (Fatemi and Folsom, 2015; Sundberg and Sahin, 2015; Varghese et al., 2017). Recent studies suggest that PC dysfunction caused by mutations in *Tsc1, Tsc2, Shank2*, and *PTEN* during a critical developmental period may contribute to behavioral deficits relevant to ASDs (Tsai et al., 2012, 2018; Reith et al., 2013; Cupolillo et al., 2016; Peter et al., 2016).

The *Scn8a* gene encodes the voltage-gated sodium channel α subunit Nav1.6. Nav1.6 is enriched at the neuronal axon initial segment and nodes of Ranvier, where it promotes neuronal excitability by participating in the initiation and propagation of action potentials (O'Brien and Meisler, 2013; Meisler et al., 2016). Na_v_1.6 is expressed throughout the central nervous system, including high expression in cerebellum, particularly in PCs (Schaller and Caldwell, 2003; Chen et al., 2009). In humans, pathogenic *SCN8A* variants are associated with a wide spectrum of phenotypes, from benign familial infantile seizures to developmental and epileptic encephalopathies (Gardella and Moller, 2019; Gertler and Carvill, 2019; Meisler, 2019). While prior work focused on the role of *SCN8A* in epileptic encephalopathies, there is evidence in humans and mice that variants in *SCN8A* are also associated with intellectual disability and neuropsychiatric abnormalities (Trudeau et al., 2006; McKinney et al., 2008; Blanchard et al., 2015; Butler et al., 2017; Wagnon et al., 2017; Liu et al., 2019; Meisler et al., 2021). Mice with heterozygous loss of *Scn8a* expression display enhanced emotionality in contextual fear conditioning, open field test and the light dark box test (McKinney et al., 2008). Conditional PC-specific *Scn8a* knockout mice were previously found to exhibit ataxia, impaired coordination, and deficits in delay eyeblink conditioning and Morris water maze tests (Levin et al., 2006; Woodruff-Pak et al., 2006) which are frequently observed in animal models with autistic features (Tsai et al., 2012; Reith et al., 2013; Piochon et al., 2014; Kloth et al., 2015; Cupolillo et al., 2016). Similar to findings in PC-specific *Tsc1* and *PTEN* knockout mice, PC-specific *Scn8a* knockout mice had a lower frequency of spontaneous firing of PC indicative of lower excitability (Raman et al., 1997; Khaliq et al., 2003; Levin et al., 2006).

Motivated by these studies implicating the PC of *Scn8a* mutant mice in ASD related traits, we used the Cre-loxP recombination system to generate conditional knockout mice in which *Scn8a* inactivation is restricted to PC. We found that PC-specific *Scn8a* knockout mice of C57BL/6J strain background exhibited late-onset cerebellar degeneration and deficits in motor coordination and social interaction, increased repetitive behavior, anxiety and abnormal activity of PC, demonstrating the specific contribution of PC to these *Scn8a*-dependent phenotypes.

## Methods

### Subjects

All animal experimental procedures were performed in accordance with the animal protocol approved by the Institutional Animal Care and Use Committee at the School of Medicine, Shandong University. *Scn8a*^*flox*/*flox*^ mice (Levin and Meisler, 2004) have been maintained on a C57BL/6J strain background since 2004 in the Meisler lab at the University of Michigan, and were imported to Shandong University in 2017. *L7/PCP2-Cre* transgenic mice (stock number: J006207) were purchased from the Model Animal Research Center of Nanjing University (Nanjing, China), where they had also been maintained on the C57BL/6J strain background. Mice were housed in a 12-h light/dark cycle (lights on 7:00 AM) with controlled temperature and humidity and ventilated with a dedicated system. All mice had *ad libitum* access to sterile food and water. We crossed *Scn8a*^*flox*/*flox*^*, L7Cre*^+^ female mice and *Scn8a*^*flox*/*flox*^,*L7Cre*^−^ male mice to generate *Scn8a*^*flox*/*flox*^,*L7Cre*^−^ (control) and *Scn8a*^*flox*/*flox*^ *L7Cre*^+^ (mutant) mice on the C57BL/6J strain background using the previously described breeding scheme (Levin et al., 2006).

### Immunohistochemistry

Mice were intraperitoneally anesthetized with 4% chloral hydrate and transcardially perfused with PBS and then 4% paraformaldehyde (PFA). Brains were extracted, postfixed overnight in 4% PFA, dehydrated, embedded in paraffin, and sectioned at 3 μm. The sections were submerged into EDTA antigenic retrieval buffer (pH 8.0) and microwaved for antigenic retrieval. Sections were blocked with 10% goat serum and 0.5% Triton X-100 in 1× PBS for 20 min. Slides were incubated in primary antibody solution overnight at 4°C. Sections were washed in 3× PBS and incubated with secondary antibody for 1 h at room temperature, then washed in 3× PBS and incubated with DAPI. Finally, anti-fluorescence quencher was used to seal the sections (Wuhan goodbio technology CO., LTD, Wuhan, China). Fluorescence images were acquired using a Nikon Eclipse Ti-SR Inverted Microscope. Images were then processed and analyzed using CaseViewer software (3D Histech Ltd, Budapest, Hungary).

The following primary antibodies were used: Calbindin (1:100; ab82812, Abcam), Na_v_1.6 (1:100; ab65166, Abcam), Caspase3 (1:250; GB11532, Wuhan goodbio technology CO., LTD, Wuhan, China). Secondary antibodies were: goat anti-mouse 488 (1:400; GB25301, Wuhan goodbio, Wuhan, China), goat anti-rabbit 488 (1:400; GB25303, Wuhan goodbio, Wuhan, China), goat anti-mouse Cy3-IgG (1:300; GB21301, Wuhan goodbio, Wuhan, China), goat anti-rabbit Cy3-IgG (1:300; GB21303, Wuhan goodbio, Wuhan, China).

### MRI Scanning Parameters

MRIs were collected on a 3-tesla MRI scanner (GE Healthcare, USA) using a standard birdcage head coil. Before the functional scans, high-resolution anatomical scans were acquired for each subject [repetition time (TR) = 2,100 ms, echo time (TE) = 111, field of view (FOV) = 10 × 10 cm, 190 slices, slice thickness = 1 mm] for later coregistration with functional maps. PD/T2 (TR = 2,500 ms, TE = 11.1/90, flip angle = 90°, FOV = 5 cm, slice thickness = 1 mm).

### Patch Clamp Recording

Six to eight week old mice were anesthetized with urethane (1.2 g/kg, i.p.) and decapitated. Parasagittal cerebellar slices were prepared with vibratome (VT 1000S, Leica, Germany) with a thickness of 300 μm. The slices were incubated in ACSF (saturated with 95% O_2_ to 5% CO_2_) for at least 1 h before recording.

After incubation, the slices were promptly transferred to the recording chamber placed on the staged of a modified upright Olympus microscope and continuously perfused with ACSF (95% O_2_ to 5% CO_2_). The patch electrodes (3–7 MΩ) were pulled on a multistage micropipette puller (P-97, Sutter Instrument, USA), and the pipette solution contained (in mM): KCl 140, MgCl_2_ 2, EGTA 10, HEPES 10, Mg-ATP 2, buffered to pH = 7.4 with KOH. After the whole-cell clamp configuration was formatted, the cells were stabilized for 5 min before recording. Then the PCs were depolarized by current steps to evoke action potential at a holding potential of −70 mV.

### TUNEL Staining Assay

Terminal deoxynucleotidyl transferase dUTP nick end labeling (TUNEL) staining was carried out on cerebellar slices according to the manufacturer's instructions (Roche). TUNEL-positive and DAPI-positive nuclei were examined using a fluorescence light inverted microscope (Nikon Eclipse TI-SR). The ratio of TUNEL-positive to total DAPI positive cells was calculated in six visual fields at ×100 magnification.

### Behavioral Tests

All behavioral tests were performed during the light cycle between 07:00 and 19:00. Male and female 5–9-week old mice were used. Similar numbers of male and female mice for each genotype were included. It is possible that genotype effects may have been underestimated or overlooked if they were sex dependent or if the baseline differences between female and male mice increased the variance in the data. Therefore, analysis of variance (ANOVA) models was used to test for the sex dependence of the genotype effects. A three-way repeated measures ANOVA with between-subject factors for genotype and sex and a repeated measure for training day was applied to accelerating rotarod and water-T maze data; a three-way ANOVA with factors for genotype, sex and pairing group was used for three-chambered test, and a two-way ANOVA with factors for genotype and sex was utilized for open-field test, light-dark transition box, elevated plus maze, grooming and male-female interaction. These analyses did not show any measures in which there was a significant effect of sex or a sex-genotype interaction. All behavioral assays were performed with the examiner blind to mouse genotypes. All the videos were analyzed by Smart software (Pan Lab, Harvard Apparatus).

### Motor Function

#### Gait Analysis

The gait of *Scn8a*^*flox*/*flox*^ *L7Cre*^+^ mutant mice was compared with *Scn8a*^*flox*/*flox*^ *L7Cre*^−^ control mice by footprint analysis as previously described (Carter et al., 1999). Briefly, to obtain footprints the fore and hind paws of the mice were coated with red and black non-toxic, water-soluble paint, respectively. Footprint patterns were analyzed using a runway (50 cm × 9 cm wide) with white paper on the bottom. The average length and width of the steps were measured.

#### Rotarod Test

Motor coordination and balance were tested with the accelerating rotarod (Panlab, Harvard apparatus) as described previously (Buitrago et al., 2004). Animals were tested over 5 consecutive days, each day consisting of 3–5 trials. The mice were placed on a 3 cm diameter rod which began rotating at 4 rpm and accelerated to 40 rpm over a period of 2 min. Latency to fall was recorded. Animals were tested at 5–6 weeks of age.

### Locomotor Activity and Anxiety

#### Open Field Test

Exploratory locomotor activity was measured in an open field as previously described (Burne et al., 2005). Each mouse was placed in an opaque open field (30 cm × 30 cm × 30 cm), under dim light. Mice were placed in the chamber for a 15 min period. Distance traveled in 1 min time bins was recorded using a centrally placed video camera and automated video tracking software (Smart software, Pan Lab/Harvard Apparatus). To assess anxiety-related behaviors, the number of entries in the center zone and percent of time in the center of the chamber was also recorded (Bailey and Crawley, 2009). Measurements were taken from animals aged 6 weeks.

#### Elevated Plus-Maze Tests

The elevated plus maze is a plus-shaped apparatus consisted of two open arms 8 × 25 cm and two closed arms (8 × 25 × 25 cm) with 8 × 8 cm central area, elevated 50 cm from the floor. Mice were placed in the central area facing one of the open arms, and allowed to freely explore the maze for 5 min. The number of entries and time spent in open or enclosed arms was measured as a parameter of anxiety-like behavior using an overhead camera and tracking system (SMART® Panlab, Harvard Apparatus).

#### Light-Dark Box Test

To further measure anxiety-like responses, the light-dark box test was performed as described previously (Tang et al., 2017). The light/dark box was constructed of plexiglass (45 × 27 × 27 cm) consisting of two chambers, a black chamber (18 × 27 cm) and a light chamber (27 × 27 cm). Mice were placed into the dark box and allowed to freely move between the light box and dark box for 5 min. The amount of time spent in the dark side and the total number of transitions between the light and dark sides were recorded.

### Social Behaviors

#### Social Interaction

The automated three-chambered social approach task is commonly employed as a standard test for assaying sociability in mice (Yang et al., 2011). The apparatus consists of a rectangular, three-chambered box made from clear polycarbonate. Retractable doorways within the two dividing walls allowed access to the side chambers. The number of entries and time spent in the chambers were automatically recorded using an overhead camera and tracking system (Smart software, Pan Lab/Harvard Apparatus). The subject mouse was allowed to habituate in the apparatus for 20 min before the sociability test, first for 10 min in the central chamber, followed by 10 min of free exploration in the entire empty arena with both doors open. In the social interaction testing period, a novel object (an inverted wire mesh cup) was placed in one of the side chambers and a novel mouse (with different genetic background matched to the subject mouse by sex and age) was placed inside an identical inverted wire cup in the other side chamber. In the social novelty testing period, another novel mouse was placed inside the empty wire cup. The apparatus was cleaned with 70% ethanol and water between subjects. Time spent interacting with the novel animal and with the object was recorded by an examiner with a stopwatch (Crawley, 2007). Animals were tested between 7 and 9 weeks of age.

#### Olfaction

We evaluated the ability of the mouse to detect novel odors and social odors as previously described (Yang et al., 2011; Tsai et al., 2012). Animals were placed in an empty, clean observation cage containing a thin layer of clean bedding and a hole on the flat filter top lid for inserting a cotton-tipped swab. Mice were habituated for 30 min with a clean cotton swab and then presented sequentially with non-social odors and social odors. Odors were presented in three consecutive trials per odorant stimulus (2 min per trial) in the following order: water, almond extract (1:100), banana extract (1:100), social odor 1, social odor 2. Social odors were created by swabbing the cotton tip in a zigzag fashion in previously soiled bedding from cages containing unfamiliar gender and age-matched animals the experimental animal had not interacted with. Time spent sniffing the swab with each presentation for each 2 min trial was measured by an investigator with a stopwatch. Measurements were taken from animals aged 7–8 weeks.

#### Male-Female Interaction

The procedure was adapted from a previously described protocol (Cupolillo et al., 2016). The test for male-female interaction was performed in a clean testing cage (Plexiglass box, 25 × 40 × 18.5 cm). Each male mouse was habituated to the testing cage for 15 min, after which an unfamiliar female of the same genotype was placed into the testing cage with a single layer of corncob bedding. An experimenter blind to the mouse genotype measured the cumulative time (by means of a stopwatch) that the male mouse spent in close contact with the female. Social interaction behavior included close following at the same speed behind the female, touching, nose-to-nose sniffing, anogenital sniffing and/or mouthing and licking the fur of the female. The cumulative time was measured (using a stopwatch) by the investigator and calculated as total time spent in contact. Animals were tested between 7 and 8 weeks of age.

### Repetitive Behavior and Reversal Learning

#### Grooming

Mice were scored for spontaneous repetitive self-grooming behavior as previously described (Silverman et al., 2010a). Each mouse was placed individually into a standard mouse shoebox observation cage with no bedding and a flat filter top lid. After habituation for 10 min, animals were observed for another 10 min. Two mice were scored simultaneously by a trained observer, who was blind to mouse genotype. Cumulative grooming time in the observation period was recorded using stopwatches. Measurements were taken from animals aged 5–6 weeks.

#### Water T-Maze

To measure reversal learning, the water T-maze was performed as described (Bednar et al., 2002; Tsai et al., 2018). A transparent platform submerged about 1 cm below the surface of the water at one of the short arms of the T-maze and served as an escape for the animals. After 1 day consisting of a habituation swim trial (60 s) with no platform present, mice were given 15 trials a day for 3 consecutive days to learn the location of the platform. After 15 trials on day 4, the platform was changed to the other short arm of the maze. Mice were then tested for 15 additional trials (reversal day 1). Then for 2 subsequent days (reversal days 2 and 3), mice were given 15 trials per day. The number of correct trials and the number of trials required to achieve five consecutive correct trials were recorded. Measurements were taken from animals aged 8–9 weeks.

### Statistical Analysis

Data are expressed as mean ± s.e.m., and statistical analysis was carried out using GraphPad Prism 8 software (GraphPad Software Inc., La Jolla, CA, USA). Statistical analyses included Student's *T*-test (paired or unpaired), one-way ANOVA followed by Tukey's *post-hoc* analysis, two-way repeated measures ANOVA followed by Bonferroni's *post-hoc* analysis. *P* < 0.05 was considered statistically significant.

## Results

### Abnormal Morphology of *Scn8a* Mutant Cerebellum

We generated Purkinje cell specific *Scn8a* knockout mice of genotype *Scn8a*^*flox*/*flox*^*,Cre*^+^ (PC *Scn8a* mutant mice) according to the breeding scheme (Levin et al., 2006). As shown in Supplementary Figure S1, PC *Scn8a* mutant mice had reduced expression of Nav 1.6 in Purkinje cells compared with control mice, whereas expression remained high in granule cells and neurons (stellate cells and basket cells) of the molecular layer in PC mutant mice.

**Figure 1:**
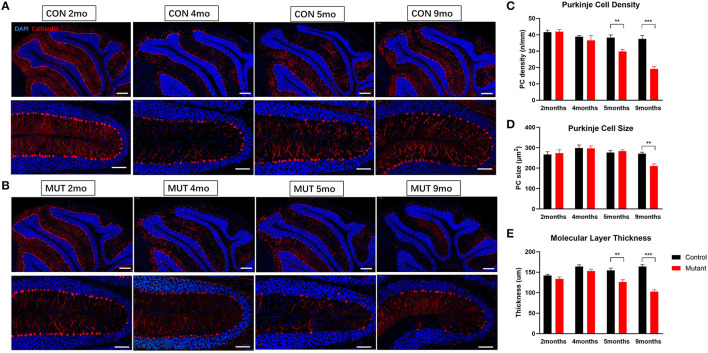
PC *Scn8a* mutant mice exhibit histological changes in cerebellum. **(A,B)** Abnormal PC morphology and gradual reduced PC number and molecular layer thickness in Scn8a mutant cerebellum (Scale bar upper row 200 μm. bottom row 100 μm). **(C,D)** The quantification of PC density and the quantification of PC soma area was performed in the IV–V. PC loss was not apparent until 5 months of age, and showed ongoing loss till 9 months **(C)**. At 9 months, mutant Purkinje cell size decreased significantly **(D)**. **(E)** The thickness of the molecular layer (ML) was normal in mutant cerebellum at 4 months of age, whereas it decreased afterward at 5 months (*n* ≥ 3 per group. Data shown are means ± SEM. **P < 0.01, ***P < 0.001, two-way ANOVA, Bonferroni's *post-hoc* analysis).

**Figure 2:**
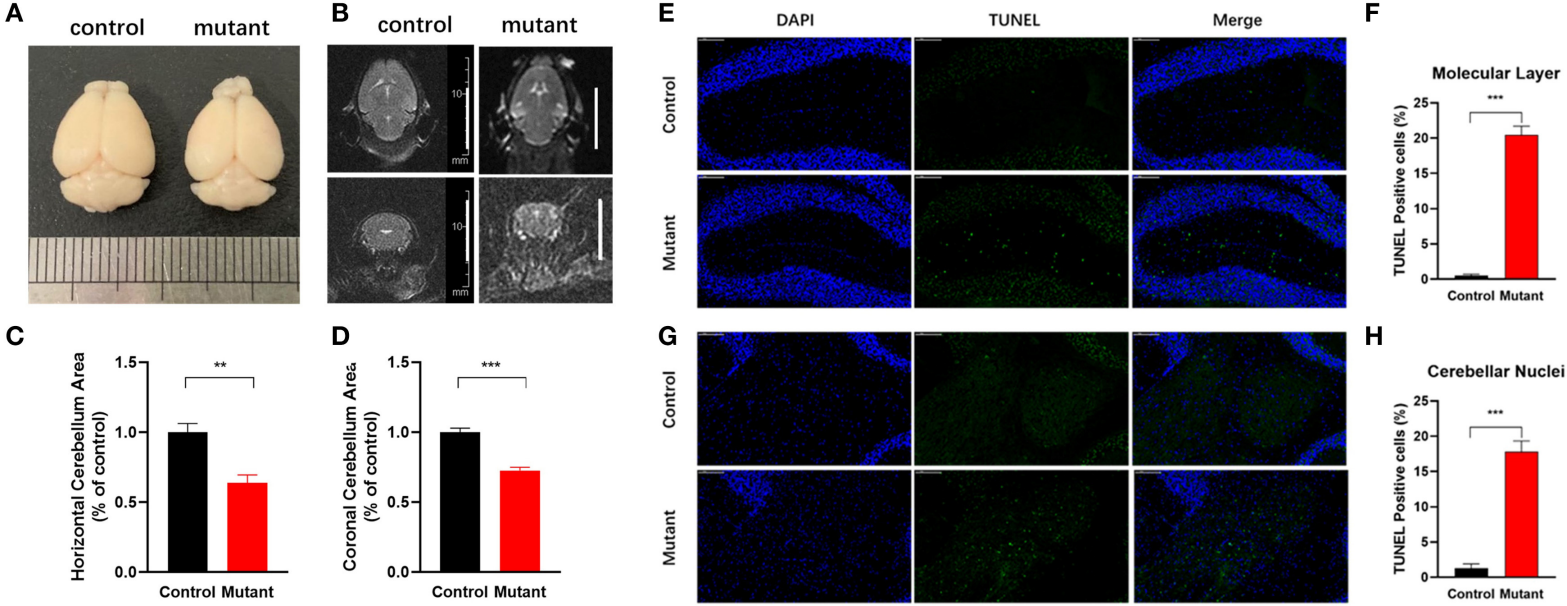
Reduced cerebellar volume and increased apoptosis in *Scn8a* PC mutant cerebellum. **(A)** Representative whole-mount images of control and PC *Scn8a* mutant brains at 9 months of age. The mutant cerebellum appears smaller than control. **(B)** Magnetic resonance imaging T2 axial and coronal images of the brain showing cerebellar volume loss in the mutant mice at 9–10 months of age (scale bar 10 mm). **(C,D)** Measurement of the cerebellar area of *Scn8a* mutant and control mice using T2-weighted 3.0 T MR imaging. Quantitative analysis revealed a significant reduction in cerebellar area in mutant animals in both axial **(C)** and coronal area **(D)** (Mean ± SEM; *n* = 5 per group; **P < 0.01, ***P < 0.001, Student's unpaired *t*-test). **(E,G)** Sections of cerebellum of control and PC *Scn8a* mutant mice at 9–10 months of age labeled with TUNEL showing apoptosis in the molecular cell layer and cerebellar nuclei (Scale bar: 100 μm). **(F,H)** Percentage of TUNEL-positive cells per unit area of cerebellar molecular layer and deep cerebellar nuclei in the PC *Scn8a* mice compared with controls (*n* = 4 for each group, ***P < 0.001, Student's unpaired *t*-test).

Consistent with earlier reports (Sprunger et al., 1999; Levin et al., 2006), cerebellar malformations were not detected in both 2 and 4-month-old PC *Scn8a* mutant mice. Calbindin staining indicated that the Purkinje cells in the mutant mice retained the regular orientation of dendrites present in control animals (Supplementary Figure S2). However, calbindin staining was noted in the granule cell layer of PC *Scn8a* mutant mice (Supplementary Figure S2, arrows). This pattern of labeling has previously been described in *Scn8a*^*med*−*jo*^ (A1071T) mice (Dick et al., 1985) and *Scn8a*^*flox*/*flox*^,*L7Cre*^+^ mice (Levin et al., 2006) and may reflect axonal swelling. We revealed that PC density, size of soma, and thickness of the molecular layer were normal at both 2 and 4 months of age (Figure 1). However, by 5 months of age, there was a reduction in the density of Purkinje cells and the thickness of molecular cell (*P* < 0.01), but the size of Purkinje cells retained normal. At 9 months of age there was a significant reduction in the thicknesses of the molecular layer and both the size and the density of Purkinje cells was significantly reduced (*P* < 0.001. *n* > 3 per group, two-way ANOVA, Bonferroni's *post-hoc* analysis). Overall brain volume was decreased at 9 months of age, as evident in the whole-mount brain (Figure 2A). MRI measurements revealed reduced area of the cerebellum of the mutant mice in both axial and coronal views (Figures 2B–D).

### Increased Apoptosis in the Cerebellum of *Scn8a* PC Mutant Mice

To determine whether neurodegeneration could account for the observed cerebellar atrophy, we adopted TUNEL staining to quantify apoptosis. The number of TUNEL-positive cells was significantly increased in PC *Scn8a* mutant mice in both the molecular layer (Figures 2E,F) and cerebellar deep nuclei (Figures 2G,H) (*P* < 0.001; *n* = 4 for mutants and control, unpaired student's *t*-test).

### Reduced Excitability in *Scn8a* Mutant PCs

It was previously demonstrated that partial or complete loss of Nav1.6 from cerebellar Purkinje cells reduces excitability and repetitive firing (Raman et al., 1997; Khaliq et al., 2003; Levin et al., 2006). To examine excitability of PCs in our mice, evoked APs were recorded by whole-cell patch clamp. Repetitive firing spikes were evoked by current injection of 200 pA with 500 ms duration. The frequency of repetitive firing was inhibited in *Scn8a* mutant PCs compared with that in control (Supplementary Figures S3A,B). The mean frequency of repetitive firing at each current injection from 50 to 400 pA was significantly reduced in *Scn8a* mutant PCs (Supplementary Figure S3C). These results confirm the previous evidence that loss of *Scn8a* inhibits the excitability of PCs (Levin et al., 2006).

### Impaired Motor Coordination in PC *Scn8a* Mutant Mice

**Figure 3:**
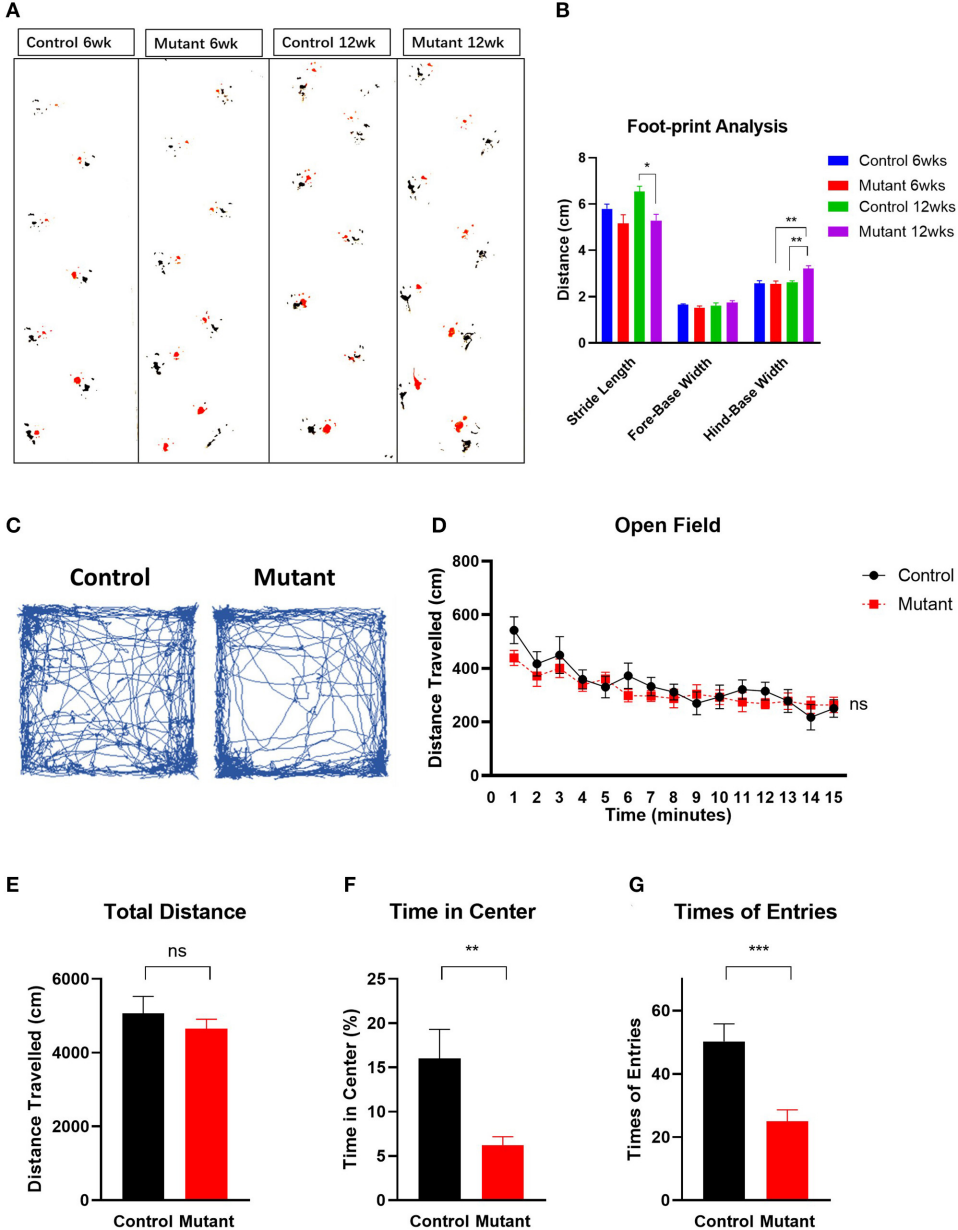
Motor coordination impairment and locomotor activity in PC *Scn8a* mutant mice. **(A)** Representative walking footprint patterns. **(B)** Gait footprint analysis at 6 and 12 weeks was evaluated for stride length, fore-base width and hind-base width. Mutant mice displayed ataxic gait with reduced stride length and increased stride width at 12 weeks of age (*n* > 6 per group. *P < 0.05, **P < 0.01; ***P < 0.001, two-way ANOVA, Bonferroni's *post-hoc* analysis). **(C)** Representative traces of mouse activity in an open field during a 15-min recording time. **(D)** Locomotor activity in the open field was comparable between control (*n* = 9) and mutant mice (*n* = 11) in a 15 min time bin (*P* = 0.4223). Time in minutes is shown on the X-axis. **(E)** The total distance moved in the open field test was not significantly different between controls and mutants (*P* > 0.05). **(F,G)** Mutant mice spent less time **(F)** in the center of the open field (*P* = 0.0063), and entered the center of the open field less often **(G)** than the controls (*P* < 0.001). Data shown are means ± SEM. ns, not significant. *P < 0.05, **P < 0.01, ***P < 0.001, two-way ANOVA, Bonferroni's *post-hoc* analysis for **(D)**. Student unpaired *t*-test for **(E–G)**.

By 6–8 weeks of age, PC *Scn8a* mutant mice developed a mildly ataxic, waddling gait that appeared to be caused by poor hindlimb coordination. However, at 6 weeks of age, mutant and control mice had comparable fore-base width and no difference in stride length and hind-base width (Figures 3A,B) (*P* > 0.05, Student's unpaired *t*-test, control 6wk *n* = 10, mutant 6wk *n* = 10). However, at 12 weeks of age, PC *Scn8a* mutant mice displayed a narrower stride length (*P* = 0.022) and a wider hind-base width (*P* = 0.007) (control *n* = 6, mutant *n* = 9), demonstrating a deficit in motor coordination.

Consistent with previous work (Levin et al., 2006), mutant mice demonstrated impaired performance on the rotarod test (Supplementary Figure S4). At 5 weeks of age, the latency to fall was significantly different between wildtype and mutant mice [two-way repeated measures ANOVA *F*_(1,18)_ = 27.99, Bonferroni *post-hoc, P* < 0.0001, *n* = 9 for control, *n* = 11 for mutant]. While control mice remained on the rod longer during each of 5 daily sessions of training, mutant mice had shorter latency to fall on day 1 (*P* < 0.001) and did not improve with time. These findings further demonstrate impaired motor coordination and poor motor learning in mice with ongoing PC loss.

### Increased Anxiety-Like Behavior in PC *Scn8a* Mutant Mice

Patients harboring loss of function variants of *Scn8a* exhibit neuropsychological abnormalities including emotional instability, anxiety and attention deficit hyperactivity disorders (Trudeau et al., 2006; Wagnon et al., 2017). We therefore carried out behavioral testing of 6-week-old PC *Scn8a* mutant mice to assess their anxiety level.

The open field exploration test is a behavioral assay widely used to evaluate locomotor responses to novel environments in rodents. Representative tracks are shown in Figure 3C. During a 15-min test, the distance traveled by mutant mice in the open field during a 1 min time bin was similar to controls [Figure 3D, two-way ANOVA, Bonferroni's *post-hoc* analysis, *F*_(1,17)_ = 0.6760, *P* = 0.4223]. The total distance traveled was also similar in controls and mutants [Figure 3E, Student unpaired *t*-test, *t*_(17)_ = 0.822, *P* > 0.05]. However, PC *Scn8a* mutants spent significantly less time in the center of the open field [Figure 3F, Student unpaired *t*-test, *t*_(17)_ = 3.254, *P* = 0.0047] and had fewer entries into the center [Figure 3G, Student unpaired *t*-test, *t*_(17)_ = 3.982, *P* < 0.001] (control *n* = 8, mutant *n* = 11).

**Figure 4:**
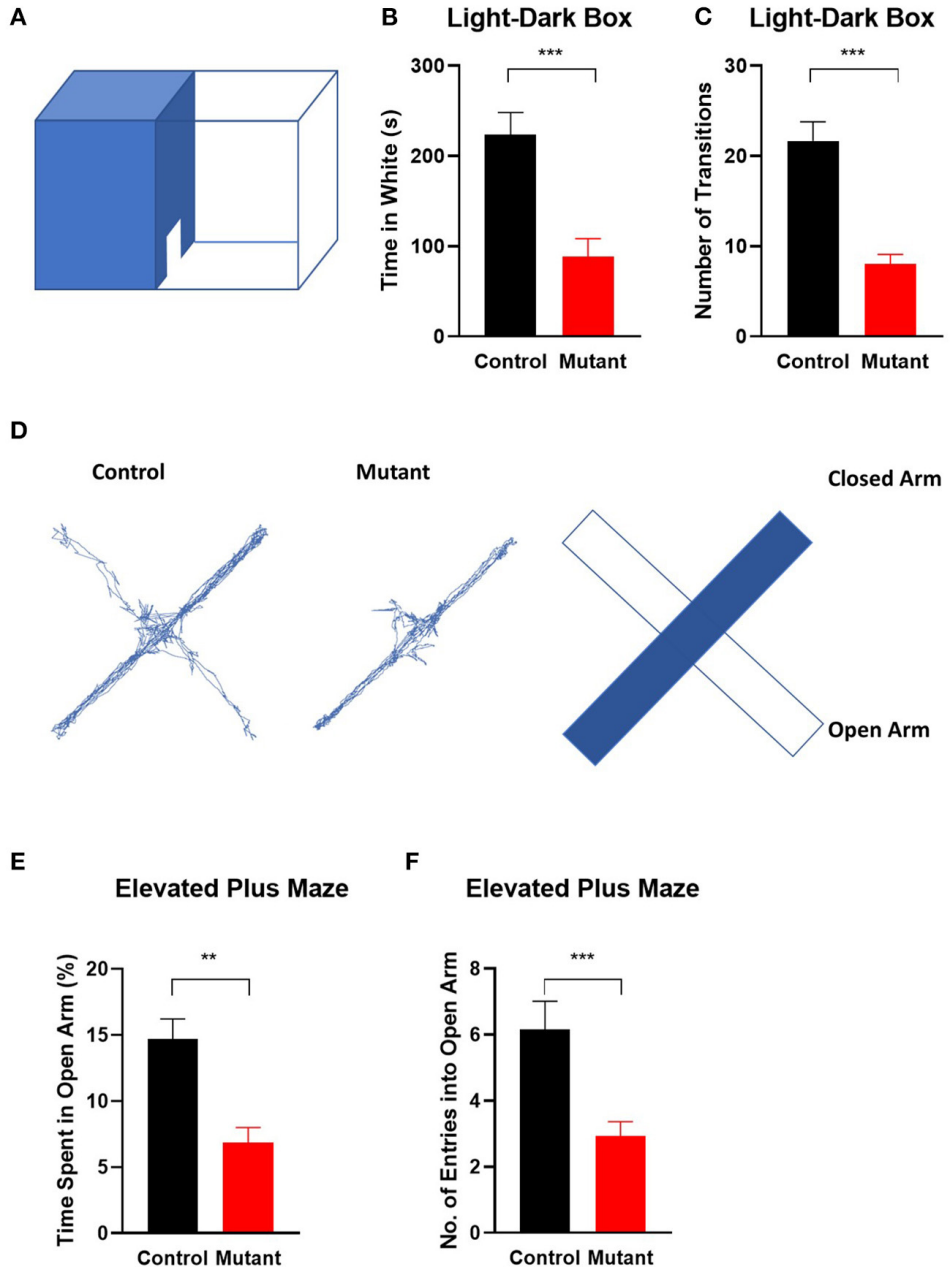
Elevation of anxiety in *Scn8a* PC mutant mice. **(A)** Schematic of light-dark transition box. In the light–dark preference test, PC *Scn8a* mutant mice spent significantly less time in the light room **(B)** and entered it less often than controls **(C)**. (*n* = 15 for control and *n* = 13 for mutant). **(D)** Representative elevated plus maze tracking plots and diagram illustrates locations of open and closed arms of the maze. Mutant mice demonstrated decreased time spent in the open arms **(E)**, and less frequently enter into the open arms **(F)** of the elevated plus maze (Control: *n* = 13 Mutant: *n* = 16). **P < 0.01, ***P < 0.001. All *P*-values by the Student's unpaired *t*-test.

As an independent test of anxiety, we used the light/dark box transition task (Bourin and Hascoet, 2003). The number of entries into the light compartment [*t*_(25)_ = 5.297, *P* < 0.0001] and the time spent in the bright area [*t*_(25)_ = 4.129, *P* < 0.001] were significantly decreased in PC *Scn8a* mutant mice, indicating increased innate anxiety-like behavior (control *n* = 15, mutant *n* = 13 Student's unpaired *t*-test) (Figures 4A–C).

To further assess anxiety, we analyzed behavior on an elevated plus maze (Hogg, 1996) (Figures 4D–F). As in the open field, mutant mice demonstrated decreased total time spent in the open arms [*t*_(27)_ = 4.191, *P* < 0.001], and a reduced number of entries into the open arms of the elevated plus maze [*t*_(27)_ = 3.532, *P* = 0.0015] (control *n* = 13 and mutant *n* = 16, Student's unpaired *t*-test).

Taken together, these assays indicate that mutant mice display increased anxiety compared with control littermates.

### Altered Social and Repetitive Behaviors in PC *Scn8a* Mice

**Figure 5:**
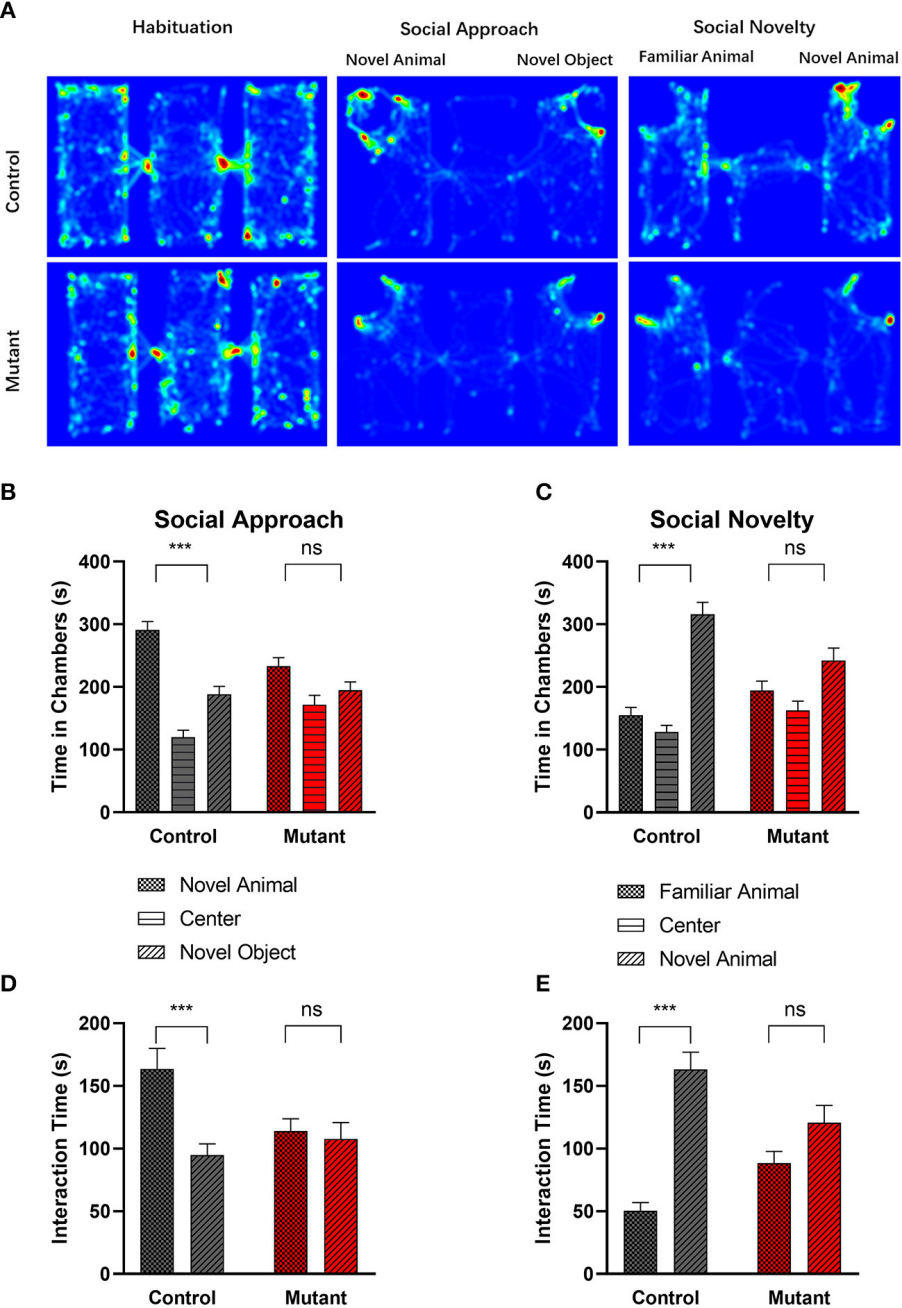
Abnormal social behavior in PC *Scn8a* mutant mice. **(A)** Representative heat maps showing time spent by Control mice (upper panel) and PC *Scn8a* mutant mice (lower panel) at each location of the three-chambered apparatus during the test. **(B,D)** In the social approach test, WT mice spent more time in the chamber with the stranger animal than in the chamber with the inanimate object **(B)**, and spent more time interacting with novel animal **(D)** in comparison with a novel object, whereas PC *Scn8a* mutant mice show no preference. **(C,E)** PC *Scn8a* mutant mice show no preference for the chamber with the novel animal in comparison with that with a familiar animal in an assay of social novelty **(C)**, and spent similar time interacting with both the novel and familiar animals **(E)**. This is in distinct contrast to control mice. Control: *n* = 16; Mutants: *n* = 17, ***P < 0.001, two-way ANOVA, Bonferroni's *post-hoc* analysis.

A three-chambered apparatus was used to measure social approach and social novelty (Figure 5). Compared with control mice, PC *Scn8a* mutant mice spent less time in the side chamber with the novel mouse and more time with the novel object (Figure 5B, *P* > 0.05). Control mice spent significantly more time with the novel mouse than with the novel object [*P* < 0.001, chamber × genotype Interaction *F*_(1,31)_ = 3.984, *P* = 0.0548, chamber effect *F*_(1,31)_ = 19.10, *P* = 0.0001, genotype effect *F*_(1,31)_ = 7.637, *P* = 0.0095, *n* = 16 for control, *n* = 17 for mutant, two-way repeated measures ANOVA, Bonferroni's *post-hoc* analysis].

We examined the amount of time each experimental mouse spent interacting with the novel mouse or the object through sniffing [Figure 5D, Genotype × chamber interaction, *F*_(1,31)_ = 7.420, *P* = 0.010; Chamber effect *F*_(1,31)_ = 11.17, *P* = 0.0022; Genotype effect *F*_(1,31)_ = 1.035, *P* = 0.3168, two-way repeated measures ANOVA, Bonferroni's *post-hoc* analysis]. While control mice showed more interest in interacting with the novel mouse (*P* < 0.001), mutant mice spent a comparable amount of time sniffing the novel mouse and the novel object (*P* > 0.05).

When the inanimate object was replaced with another novel mouse, control mice spent more time in the chamber containing a novel mouse than in the chamber with the familiar mouse (*P* < 0.001), demonstrating normal sociability [genotype × chamber interaction *F*_(1,31)_ = 6.596, *P* = 0.0153, Chamber effect *F*_(1,31)_ = 22.62, *P* < 0.0001, Genotype effect *F*_(1,31)_ = 3.479, *P* = 0.0717, two-way repeated measures ANOVA, Bonferroni's *post-hoc* analysis] (Figure 5C). In contrast, the PC *Scn8a* mutants failed to show any preference between the two social chambers (*P* > 0.05). Control mice also spent more time sniffing the novel animal over the familiar animal (Figure 5E, *P* < 0.001), while mutant mice failed to show such preference (*P* > 0.05) [genotype × chamber interaction *F*_(1,31)_ = 9.777, *P* = 0.0038, chamber effect *F*_(1,31)_ = 33.51, *P* < 0.0001, genotype effect *F*_(1,31)_ = 0.02699, *P* = 0.8706, two-way repeated measures ANOVA, Bonferroni's *post-hoc* analysis].

**Figure 6:**
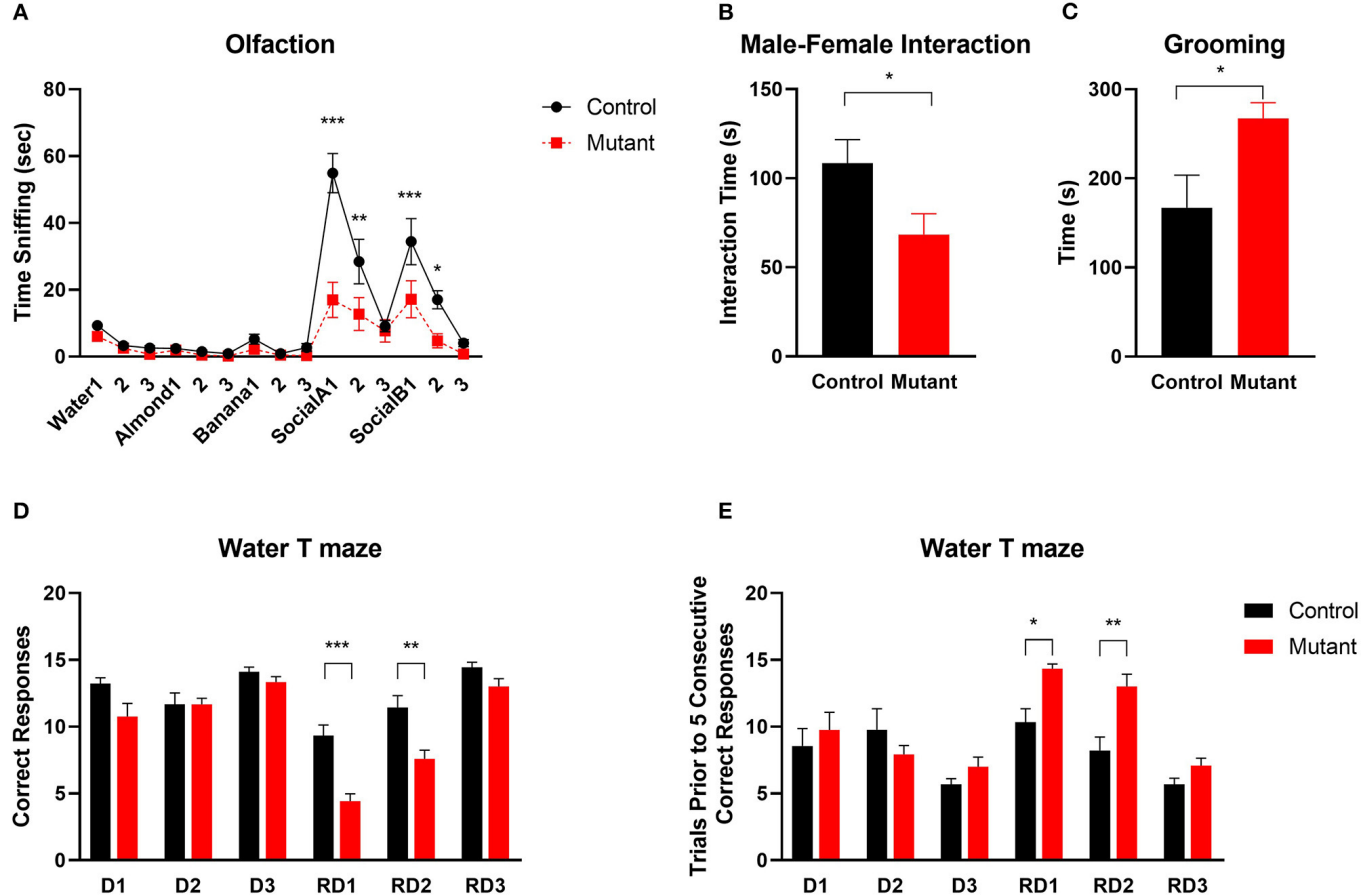
Abnormal social and repetitive behaviors and impaired reversal learning in PC *Scn8a* mutant mice. **(A)** Mutant mice spent comparable time sniffing non-social stimuli whereas less time sniffing social odor cues (*n* = 11 per group). **(B)** Reduced sociability of KO mice is indicated by reduced time interacting with a female conspecific (Control: *n* = 8; Mutant: *n* = 10). **(C)** PC *Scn8a* mutant mice spent more time self-grooming (Control: *n* = 10; Mutant: *n* = 11). **(D,E)** On Day 1 to Day 3, mutants display normal acquisition learning of the escape platform location in the water T maze as indicated by total correct trials **(D)** and trials needed for 5 consecutive correct responses **(E)**. However, on reversal day 1 and day 2, PC *Scn8a* mutant mice have significantly fewer correct trials **(D)**, and take more trials to achieve five consecutive correct responses (Control: *n* = 9, Mutant: *n* = 12). RD, Reversal Day. Data shown are means ± SEM. *P < 0.05, **P < 0.01, ***P < 0.001, Two-way ANOVA, Bonferroni *post-hoc* analysis in **(A)**, Student's unpaired *t*-test in **(B–E)**.

It is thought that impaired discrimination of social olfactory cues may contribute to social deficits in mice (Silverman et al., 2010b). We used a social olfaction assay to assess interaction with social olfactory cues (Figure 6A). When mice were first presented with three non-social odor cues (water, almond extract and banana extract), there were no significant differences between mutant and wildtype mice (*P* > 0.05, *n* = 11 Control and 11 mutants), demonstrating normal olfactory function. When social odors (social A and social B) were applied, PC *Scn8a* mutant mice spent less time sniffing the cotton tips than control mice, suggesting a lack of interest in social odor stimuli [*n* = 11 per group, two-way repeated measures ANOVA *F*_(1,20)_ = 15.25 Bonferroni *post-hoc, P* < 0.0001 for Social A1, *P* = 0.001 for Social A2, *P* < 0.0001 for Social B1, *P* = 0.036 for Social B2].

Social behavior was further tested by assessing social interaction with a female conspecific mouse. Compared with control mice, there was a significant reduction in the time spent sniffing, allogrooming, mounting, or following the female by male PC *Scn8a* mutant mice [Student's *t*-test *t*_(18)_ = 2.23, *P* = 0.038; Control *n* = 8; Mutant *n* = 12] (Figure 6B).

One of the diagnostic behavioral symptoms of autism is repetitive behavior. We analyzed grooming as an index for stereotyped, repetitive behaviors. PC *Scn8a* mutant mice engaged in much longer bouts of self-grooming than control mice (Figure 6C) [*n* = 14 per group, Student's *t*-test *t*_(26)_ = 2.48, *P* = 0.019].

### Perseveration (Behavioral Inflexibility) in PC *Scn8a* Mutant Mice

ASD patients often show inflexible and rigid behavior and thinking (Bralten et al., 2018). To evaluate behavioral flexibility, we used the reversal water-T maze assay (Figures 6D,E). During the first 3 days of training, while the platform was in the right arm of the maze, both groups of mice performed equally well and learned the location of submerged platform. On the first reversal day with the platform moved to the left arm of the maze, PC *Scn8a* mutant mice incorrectly visited the right arm more frequently than control mice. Mutant mice also needed more trials to achieve 5 consecutive correct responses. Comparable results were observed on Reversal day 2. [Reversal Day 1, number of correct trials: Student's *t*-test *t*_(19)_ = 5.097, *P* < 0.0001; number of trials prior to five consecutive correct trials: Student's *t*-test *t*_(19)_ = 3.221, *P* = 0.0045; Reversal Day 2: number of correct trials: Student's *t*-test *t*_(19)_ = 3.48, *P* = 0.0025; number of trials prior to five consecutive correct trials: Student's *t*-test t_(19)_ = 3.467, *P* = 0.0026]. By the third reversal day, the mutant mice behaved similarly to the control mice in number of correct trials and number of trials prior to 5 consecutive correct responses.

## Discussion

The first link between human disease and *SCN8A* mutation was obtained in 2006 in a study of a small pedigree in which heterozygous carriers of a loss-of-function *SCN8A* mutation exhibited a range of phenotypes including ataxia, cognitive deficits, and emotional instability (Trudeau et al., 2006). Since the development of next generation sequencing, many pathogenic variants of *SCN8A* have been identified in patients with a spectrum of neurodevelopmental disorders, and some genotype-phenotype correlations have emerged. *SCN8A* missense variants with gain-of-function channel properties are associated with developmental epileptic encephalopathy with early onset of severe seizures, hypotonia, and paroxysmal dyskinesia. In contrast, loss of function mutations of SCN8A can cause autism or intellectual disability without seizures (Larsen et al., 2015; Gertler and Carvill, 2019; Liu et al., 2019; Meisler et al., 2021). In a recent study by Wong et al. (2021), they generated a novel *Scn8a* mouse model carrying the human R1620L mutation (with both gain- and loss-of-function effects) which exhibited a range of behavioral abnormalities, including hyperactivity, impaired learning and memory and social deficits. These findings suggest that *SCN8A* dysfunction may contribute to other neurological and neuropsychiatric disorders. Neuropsychiatric comorbidities were not previously studied in Purkinje cell specific *Scn8a* knockout model, and the role of reduced Nav1.6 in cerebellar function was incompletely characterized.

In this study, we provide evidence that *SCN8A* in cerebellar PCs has a key role in mechanisms involved in ASDs. We show that mice of C57BL/6J strain background with selective disruption of *Scn8a* in PCs display behavioral traits related to neuropsychiatric abnormalities such as ASDs and anxiety, associated with graded loss of PCs and progressive cerebellar atrophy.

One of the main symptoms of cerebellar dysfunction in humans is ataxia (Schniepp et al., 2017). Our results revealed that PC *Scn8a* mutant mice exhibit deficits in motor coordination and motor learning in the rotarod test. Gait analysis demonstrated wide base and ataxic gait. Similar features are also seen in patients with loss of function mutation of *SCN8A* (Trudeau et al., 2006), and in patients with ASD and other murine models of ASD (Fatemi et al., 2012; Tsai et al., 2012; Reith et al., 2013; Cupolillo et al., 2016).

PC *Scn8a* mutant mice were previously shown to be impaired in delay eyeblink conditioning (Woodruff-Pak et al., 2006), an additional connection to cerebellar dysfunction and autism. Eyeblink conditioning is affected in the general ASD patient population, and is viewed as a biomarker for ASD (Oristaglio et al., 2013; Welsh and Oristaglio, 2016). Eyeblink-conditioning defects appear more often in mouse autism models than in non-autism-like phenotypes (Kloth et al., 2015). Given the similarity of the PC Scn8a mutant mice to other autism models with regards to motor deficit, delay eye-blink conditioning impairment and electrophysiological changes in Purkinje cells, it is not surprising that PC *Scn8a* mutant mice also exhibit ASD-relevant social deficits.

Purkinje cell loss is the most consistent presentation in post mortem studies of ASD patients, with 35–95% fewer cerebellar Purkinje cells in ASD brains than controls (Whitney et al., 2009; Wegiel et al., 2014; Mosconi et al., 2015). Purkinje cells are recognized as key cells mediating autism-like phenotypes in mice (Fatemi et al., 2012; Tsai et al., 2012; Reith et al., 2013; Cupolillo et al., 2016). PC *Scn8a* mutant mice also displayed abnormalities in the composition of the cerebellum. The thickness of molecular layer as well as the density of Purkinje cells were comparable at early age. However, Purkinje cell beginning to lose between 4 and 5 months of age, with an ongoing and significantly decreased in both Purkinje cell density and thickness of molecular layer in PC *Scn8a* mutant mice by 9 months of age (Figure 1). Progressive PC loss is accompanied by cerebellar atrophy indicated by both gross anatomy and MR imaging of cerebellum.

Previous studies demonstrated a relationship between cerebellar function and behavior including sociability (novel mouse vs. novel object), social preference (familiar mouse vs. novel mouse), social odor preference (conspecific urine vs. water), and male-female interaction (Tsai et al., 2012; Cupolillo et al., 2016). Our demonstration of social deficits in three-chambered tests were highly consistent with earlier studies in other cerebellar genetic mouse models of autism (Tsai et al., 2012; Reith et al., 2013). PC *Scn8a* mutant mice also displayed reduced responses to female social cues in a male-female reciprocal social interaction context. Mutant mice spent significantly less time sniffing the social odors than control mice in the context of olfaction test.

Autism and anxiety disorders are frequently comorbid with each other (Adams et al., 2020; Baribeau et al., 2020, 2021), and autistic and anxious traits are highly correlated (Ha et al., 2016; Tatsukawa et al., 2019). Likewise, co-existing anxiety-like behaviors were observed in the *Scn8a* PC mutant mice. Anxiety-like behaviors in the mutant mice included reduced exploration behavior in the center in the open field test and reduced non-social anxiety in the elevated plus maze and light-dark transition test. Similar anxiety-like behaviors also presented in autistic mice with Purkinje cell-specific deficiency of Shank2 (Ha et al., 2016) and Pianp mice (Winkler et al., 2020). McKinney et al. (2008) investigated mice heterozygous for a null mutation of *Scn8a* (*Scn8a*^+/−^) and demonstrated avoidance of well-lit, open environments as well as pronounced stress-induced coping behavior. Impaired Purkinje cell firing was also demonstrated in heterozygous *Scn*8*a*^+/−^ null mice (Raman et al., 1997; Khaliq et al., 2003). Our data on the PC-specific KO of *Scn8a* suggest that the enhanced anxiety-like behaviors in *Scn8a* heterozygous null mice may be mediated by altered output signaling from the cerebellum.

It has been observed that cognitive inflexibility and cerebellar pathology co-occur in psychiatric disorders (e.g., autism, schizophrenia, addiction). Recent studies using lurcher mutant mouse, which lose 100% of their Purkinje cells postnatally or lurcher mouse chimeras, which lose varying numbers of Purkinje cells, suggested impairment in behavioral flexibility, reversal learning and increased repetitive behaviors, as well as higher level cognitive processes (Dickson et al., 2010, 2017; Martin et al., 2010). Inflexibility to change in routine can be viewed as a form of perseverative behavior, and assessments of reversal learning are used as a behavioral endpoint in studies of rodent models. In water T-maze tests, the initial acquisition of the behavior was normal but PC *Scn8a* mutant mice exhibited impaired reversal learning. Autistic patients often exhibit repetitive behaviors that refuse to change (Hollander et al., 2003)_._ This feature can be recapitulated by rodent grooming behaviors (Crawley, 2004). PC *Scn8a* mutant mice displayed increased repetitive behavior, including self-grooming. All these results, together with previous findings in lurcher mutant mouse and lucher chimeras, indicated that cerebellar pathology may play a causal role in the generation of repetitive behaviors and cognitive inflexibility.

Previous research found the levels of Bcl-2 and P53 protein, two important markers of apoptosis, were increased in cerebellum of autistic subjects compared with controls (Araghi-Niknam and Fatemi, 2003). Dong et al. (2018) demonstrated elevation of ER stress signals, oxidative stress, and apoptosis in the molecular layer of the autistic cerebellum. Deep cerebellar nuclear cells to which Purkinje cells project are abnormal in ASD, showing enlargement during childhood followed by reduction in size and number during adolescence and adulthood (Bauman, 1991). Using the TUNEL assay, we found increased cell apoptosis in molecular layer of PC *Scn8a* mutant mice. Interestingly, we also noted obvious cell apoptosis in neurons of the deep cerebellar nuclei.

In Purkinje cells lacking Nav1.6 channels, both spontaneous firing activity and high frequency discharge are impaired (Raman et al., 1997; Khaliq et al., 2003; Levin et al., 2006). We also confirmed the reduced excitability in *Scn8a* mutant PCs. Since PC provide the only output from the cerebellar cortex, the reduced firing of Purkinje cells observed in many ASD-like mouse models (Tsai et al., 2012; Cupolillo et al., 2016; Yang et al., 2020) may disinhibit the downstream deep cerebellar nuclei, which gate the outgoing information to the thalamus, basal ganglia, and neocortex (Sundberg and Sahin, 2015), potentially influencing integrative networks (Bostan and Strick, 2018). Neurons of the deep cerebellar nuclei receive major basal and driven inhibition from PC, but they are also spontaneously active, producing action potentials even without excitation (Zheng and Raman, 2011). Therefore, we suggest that loss of *Scn8a* in Purkinje cells might disrupt Purkinje-mediated inhibition and increase firing in the deep cerebellar nuclei. The resulting elevated spontaneous activity of cerebellar nuclei could result in oxidative stress and induced apoptosis. Further research is needed to elucidate the mechanism connecting Purkinje cell loss to cerebellar apoptosis.

However, it should be noted that our study was performed on a single inbred strain, C57BL/6J. Therefore, our understanding of these effects is limited to this single genome type. However, recent studies showed that results from studies that utilize diverse genetic backgrounds are a better model of complex disease across individuals and are more likely to generalize across patient populations (Neuner et al., 2019). Therefore, further research is needed to validate the effects of loss of *Scn8a* expression in Purkinje cells on cognitive and autistic behavior changes using different inbred strains.

## Conclusion

We have demonstrated that mice with loss of *Scn8a* in Purkinje cells provide a new model of features of ASD. Mice of C57BL/6J strain with homozygous loss-of-function of Nav1.6 channels in Purkinje cells exhibit motor deficits and autistic traits such as deficits in social interaction, stereotyped behaviors and anxiety-like behaviors, demonstrating an ASD-like phenotype with strong resemblance to other Purkinje cell-dependent models of ASD (Fatemi et al., 2012; Tsai et al., 2012; Reith et al., 2013; Cupolillo et al., 2016). Together with previously reports of reduced spontaneous and repetitive firing (Raman et al., 1997; Levin et al., 2006), learning deficits in the rotarod test (Levin et al., 2006) and delay eyeblink conditioning impairment (Woodruff-Pak et al., 2006), these studies support PC *Scn8a* mutant mice as a model that reproduces many symptoms of patients with *SCN8A* loss of function mutations.

Our study has demonstrated the specific contribution of loss of *Scn8a* in cerebellar Purkinje cells to behavioral deficits characteristic of ASD. These results provide novel insights into mechanisms contributing to the pathogenesis of ASDs. Future research to unravel the pathogenesis underlying molecular and cellular alterations will improve our understanding of the still enigmatic fields of ASD and anxiety, and open new avenues for molecular diagnosis and therapy.

## Data Availability Statement

The original contributions presented in the study are included in the article/Supplementary Materials, further inquiries can be directed to the corresponding author/s.

## Ethics Statement

The animal study was reviewed and approved by Ethics Committee of the School of Basic Medical Sciences of Shandong University.

## Author Contributions

XY and QL designed the experiments and synthesized and analyzed data. HY performed the electrophysiology study and analyzed data. XY, BL, and AC performed behavioral studies. AL and GZ performed and analyzed MRI data. XW, YS, and XB contributed to animal husbandry. XY and XW performed immunohistochemistry experiments and analyzed data. XY and MM organized the manuscript. DE-F, ZY, MM, and QL reviewed and revised the manuscript. All authors contributed to the article and approved the submitted version.

## Funding

This project was supported by National Natural Science Foundation of China (Grant Nos. 81601195, 81671114, 81741055, and 81873878), Natural Science Foundation of Shandong Province (Grant Nos. ZH2016HB38 and ZR2021MH229), and Open Research Fund of National Health Commission Key Laboratory of Birth Defects Prevention (ZD202101).

## Conflict of Interest

The authors declare that the research was conducted in the absence of any commercial or financial relationships that could be construed as a potential conflict of interest.

## Publisher's Note

All claims expressed in this article are solely those of the authors and do not necessarily represent those of their affiliated organizations, or those of the publisher, the editors and the reviewers. Any product that may be evaluated in this article, or claim that may be made by its manufacturer, is not guaranteed or endorsed by the publisher.

## Supplementary Material

Supplementary Figure S1 Sagittal cryosection of cerebellar Nav1.6 staining (green) and calbindin staining (red) showing the deficiency of Nav1.6 expression in PC of the *Scn8a* mutant mice. Nav1.6 staining in control Purkinje cells are indicated by yellow arrows. Scale bar 50 μm.

Supplementary Figure S2 Scattered calbindin staining in the granule cell layer along the proximal segment of PC axons of Purkinje Scn8a mutant mice (arrows). Scale bar 100 μm.

Supplementary Figure S3 Reduced excitability of *Scn8a* mutant PCs. **(A,B)** The evoked action potential in control and *Scn8a* mutant PCs in response to depolarization of 200 pA. **(C)** Comparison of the mean frequency of repetitive firing at command potentials (*n* = 10 for both control and Scn8a mutant groups. All data were presented as mean ± SEM, ^***^*P* < 0.001).

Supplementary Figure S4 Motor coordination and motor learning assessed on an accelerating rotarod. PC *Scn8a* mutants displayed decreased time on an accelerating rotarod and no improvement with training (Control: *n* = 9, Mutant: *n* = 11. *P* < 0.001, two-way ANOVA, Bonferroni's *post-hoc* analysis).
