# Supplementary figures and images for: Social Deficits and Cerebellar Degeneration in Purkinje Cell Scn8a Knockout Mice
Source: Front Mol Neurosci. 2022 Apr 26;15:822129. doi: 10.3389/fnmol.2022.822129 (PMC9087741; doi:10.3389/fnmol.2022.822129)

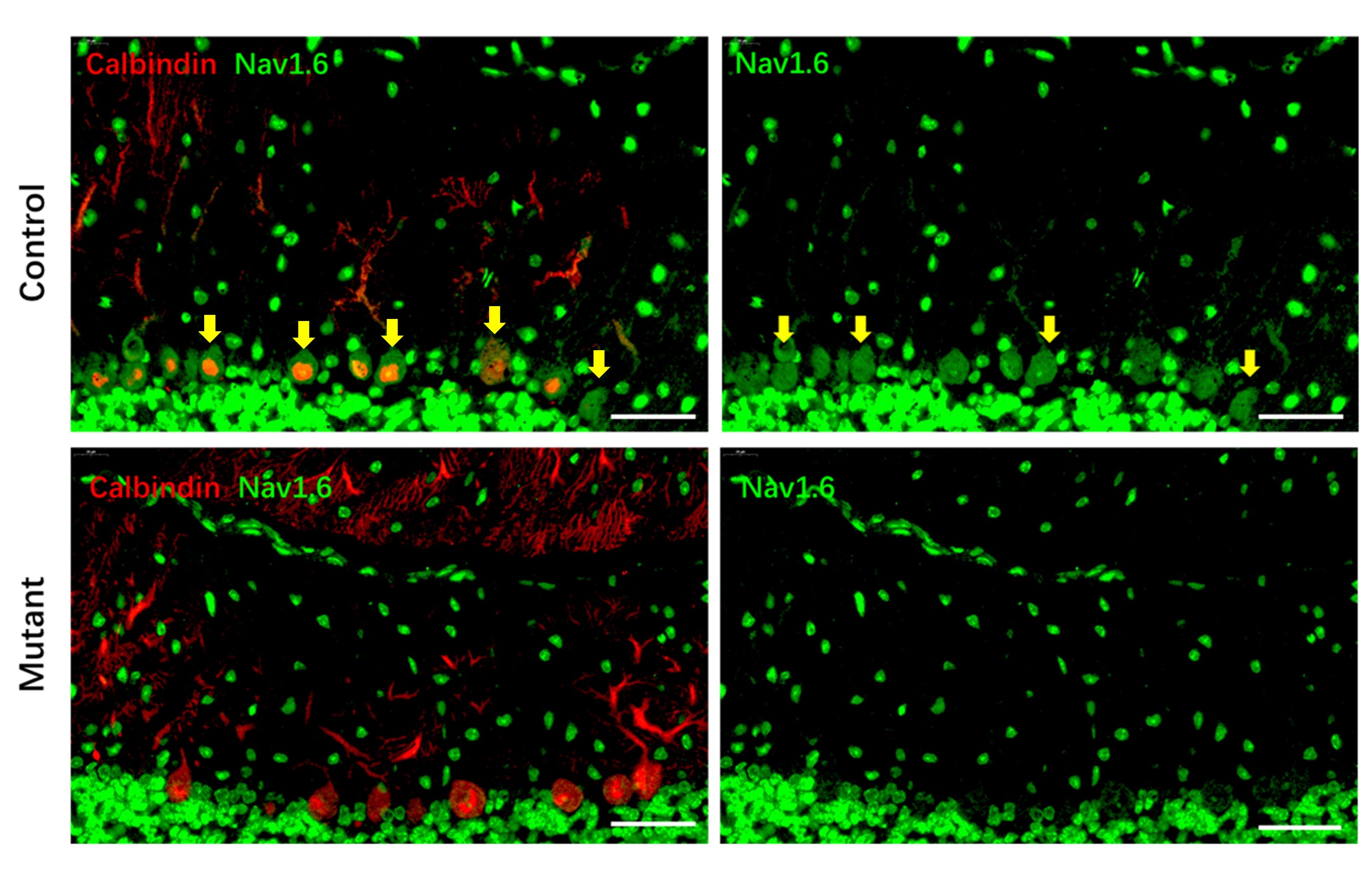

Supplement: Supplementary Figure S1 — Sagittal cryosection of cerebellar Nav1.6 staining (green) and calbindin staining (red) showing the deficiency of Nav1.6 expression in PC of the Scn8a mutant mice. Nav1.6 staining in control Purkinje cells are indicated by yellow arrows. Scale bar 50 μm. [file Image_1.JPEG]

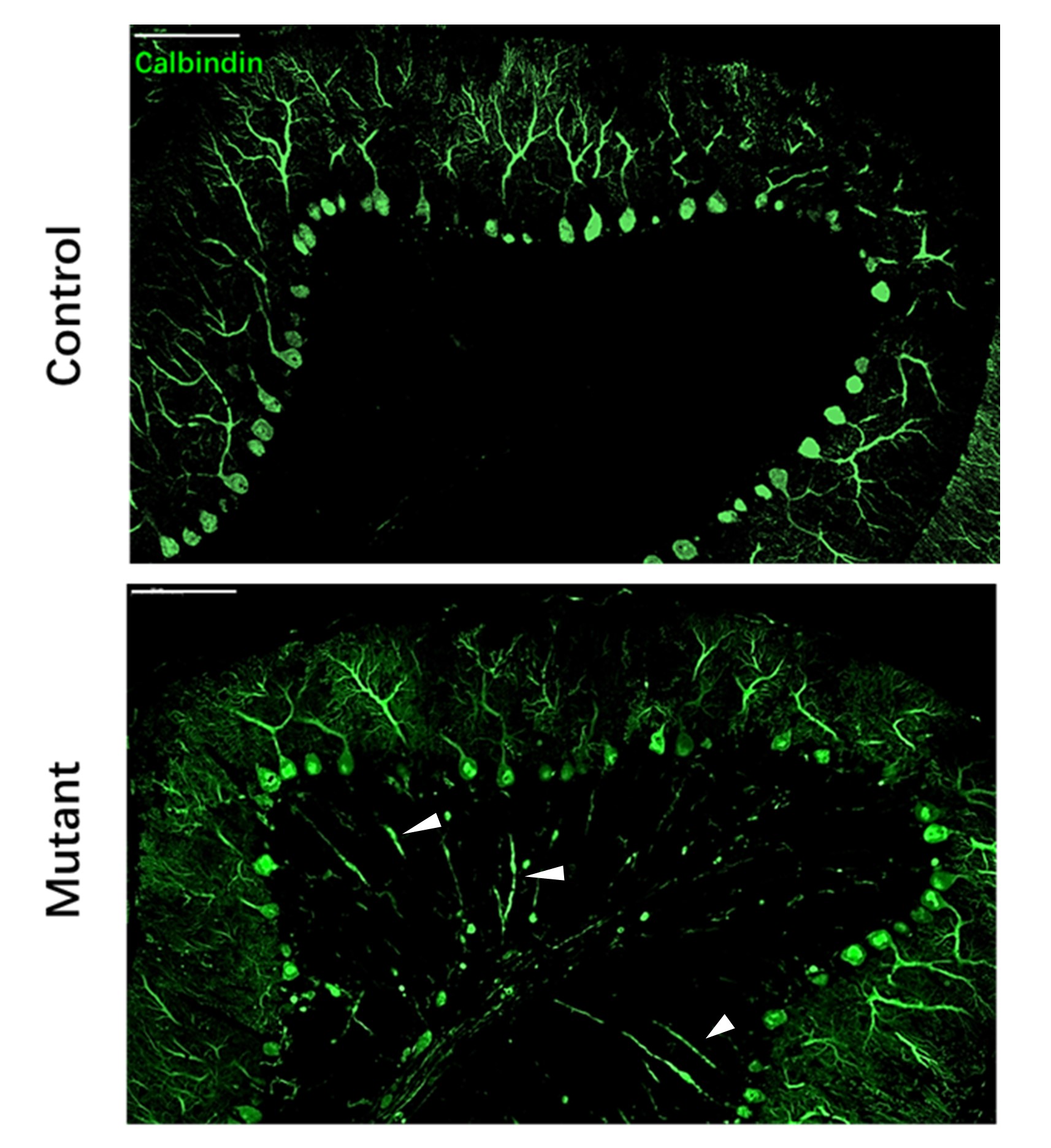

Supplement: Supplementary Figure S2 — Scattered calbindin staining in the granule cell layer along the proximal segment of PC axons of Purkinje Scn8a mutant mice (arrows). Scale bar 100 μm. [file Image_2.JPEG]

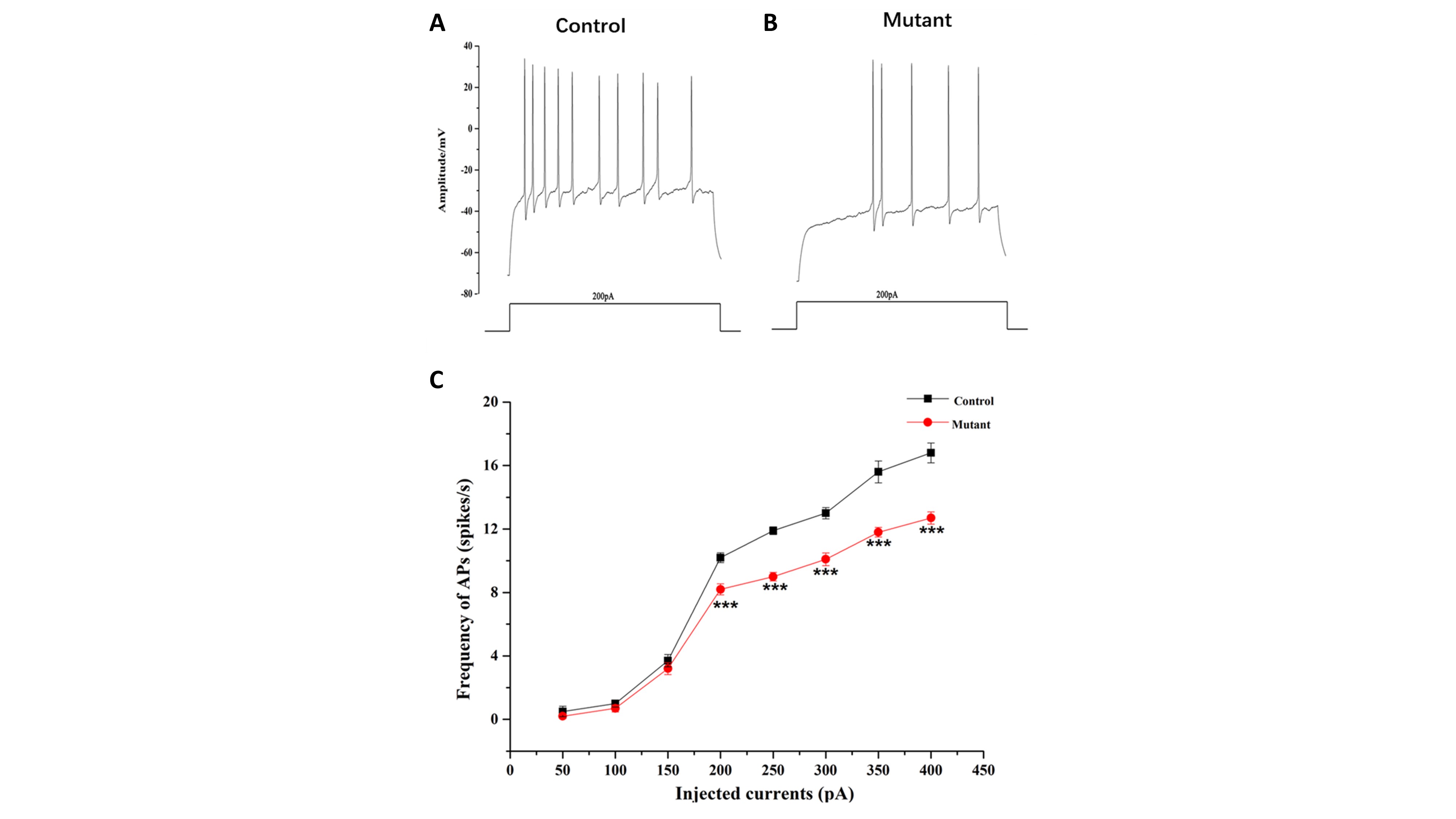

Supplement: Supplementary Figure S3 — Reduced excitability of Scn8a mutant PCs. (A,B) The evoked action potential in control and Scn8a mutant PCs in response to depolarization of 200 pA. (C) Comparison of the mean frequency of repetitive firing at command potentials (n = 10 for both control and Scn8a mutant groups. All data were presented as mean ± SEM, ***P < 0.001). [file Image_3.JPEG]

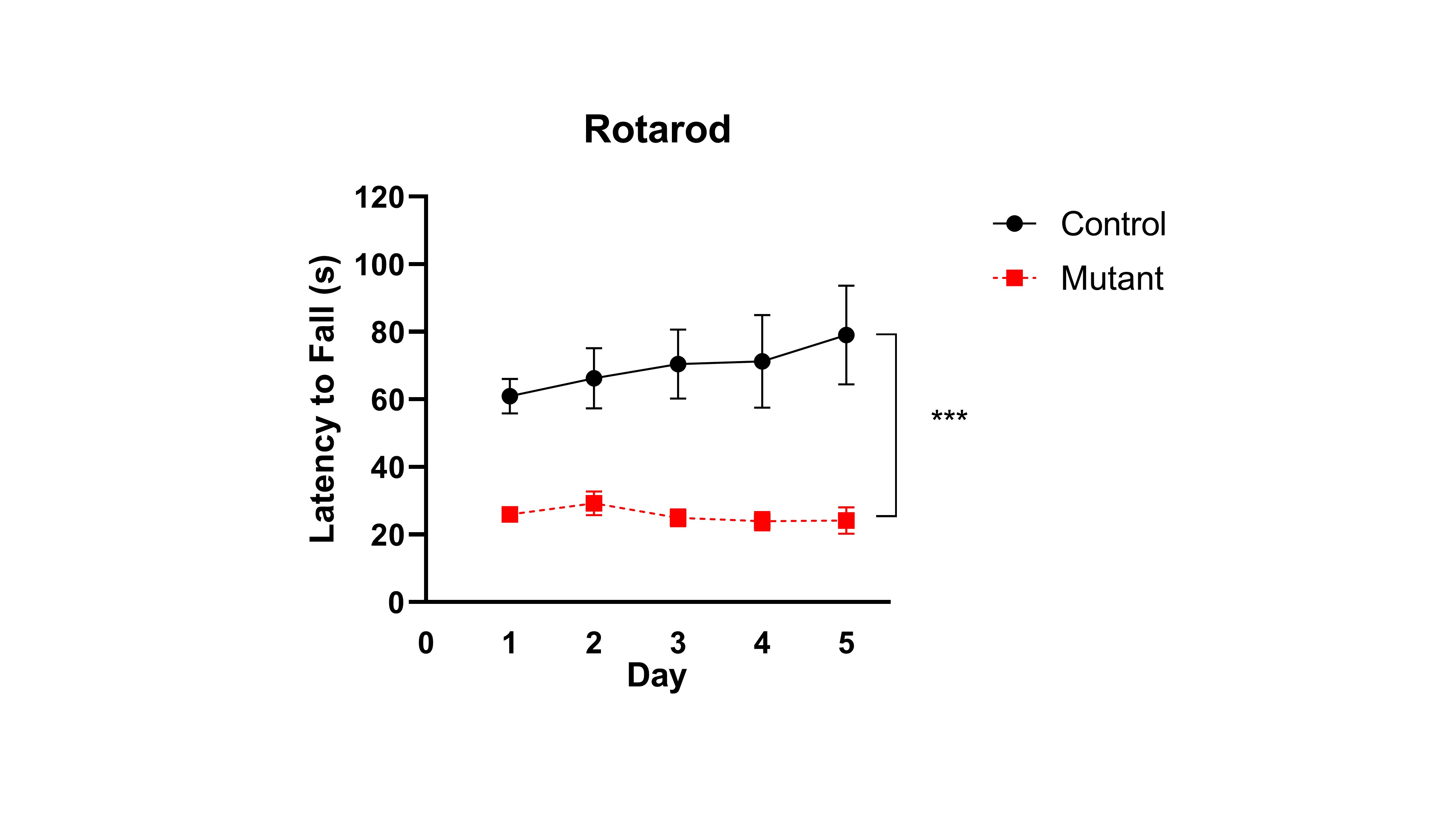

Supplement: Supplementary Figure S4 — Motor coordination and motor learning assessed on an accelerating rotarod. PC Scn8a mutants displayed decreased time on an accelerating rotarod and no improvement with training (Control: n = 9, Mutant: n = 11. P < 0.001, two-way ANOVA, Bonferroni's post-hoc analysis). [file Image_4.JPEG]
